# Corneal Astigmatism Alteration after Combined Silicone Oil Removal and Cataract Surgery with Intraocular Lens Implantation

**DOI:** 10.1155/2023/6175272

**Published:** 2023-06-28

**Authors:** Zhenyu Ji, Ting Su, Lu Li, Tao He, Yu Su

**Affiliations:** Eye Center, Renmin Hospital of Wuhan University, Wuhan, China

## Abstract

**Purpose:**

To explore short-term changes in corneal astigmatism after combined silicone oil removal and cataract (SORC) surgery.

**Methods:**

We enrolled 89 patients (43 men and 46 women). Zeiss IOLMaster was used to measure corneal astigmatism status and axial length on the day before and after the SORC surgery. Best-corrected visual acuity (BCVA) and intraocular pressure (IOP) were recorded. The results were compared to the outcomes at 3 days, 1 week, and 1 month postoperatively.

**Results:**

Compared to baseline, K1 decreased significantly at 3 days postoperatively (*P* = 0.016), 1 week (*P* = 0.009), and 1 month (*P* = 0.035), while K2 increased significantly at 3 days postoperatively (P = 0.002), 1 week (*P* < 0.001), and 1 month (*P* = 0.001), as well as corneal astigmatism (all *P* < 0.001). Compared to that at the baseline, BCVA significantly improved at 3 days, 1 week, and 1 month postoperatively (all *P* < 0.001). Meanwhile, IOP decreased significantly at 3 days postoperatively (*P* < 0.001), 1 week (*P* = 0.005), and 1 month (*P* = 0.007). Similarly, axial length decreased at all follow-up time points (all *P* < 0.001).

**Conclusion:**

Corneal astigmatism increased in the short term after the SORC operation but gradually decreased at 1 month postoperatively. BCVA improved steadily, and SORC was widely used in the clinic.

## 1. Introduction

Pars plana vitrectomy (PPV) has been highly developed for various vitreoretinal diseases [1], and transconjunctival 23G PPV has become a standard procedure [2–4]. As an important temporal vitreous tamponade during eye surgery, silicone oil has been extensively applied in the clinic for diseases such as retinal detachment and diabetic retinopathy because of its nontoxicity, good optical permeability, and large surface tension [5, 6].

Long-term silicone oil tamponade causes a series of complications, including cataracts, glaucoma, and corneal degeneration; therefore, silicone oil should be removed 3–6 months postoperatively [7–9]. Since cataract has the highest incidence among complications [10, 11], combined silicone oil removal and cataract (SORC) surgery with intraocular lens implantation is routinely considered in phakic eyes when silicone oil should be removed [12–14]. During SORC surgery, doctors can check the fundus more clearly and easily compared to simple silicone oil removal (SOR) surgery. Moreover, SORC surgery can improve postoperative visual acuity and reduce costs by avoiding secondary surgery. Therefore, a combination of SORC surgery with intraocular lens implantation is widely used.

As a common refractive error, astigmatism can cause blurred vision and visual fatigue, which directly influence quality of life. Surgically induced astigmatism (SIA), an important component of astigmatism, affects visual quality and visual rehabilitation postoperatively [15, 16]. However, previous studies have mainly focused on changes in corneal astigmatism after cataract surgery, and there are few relevant studies on SORC surgery with intraocular lens implantation.

This study aimed to explore the short-term changes in corneal astigmatism after SORC surgery to reduce postoperative astigmatism in future clinical work.

## 2. Patients and Methods

### 2.1. Patients

Eighty-nine patients (43 men and 46 women) were enrolled in the Department of Ophthalmology at Renmin Hospital of Wuhan University. Patients with rhegmatogenous retinal detachment and proliferative diabetic retinopathy who underwent SORC surgery between November 2021 and June 2022 were included in this study. Only one eye was enrolled for every indivious condition. The study conformed to the Declaration of Helsinki, and ethical approval was obtained from the Medical Ethics Committee of the Renmin Hospital of Wuhan University. All patients signed informed consent forms after being informed of the purposes, contents, and potential risks.

### 2.2. Inclusion and Exclusion Criteria

The inclusion criteria were eyes with rhegmatogenous retinal detachment and proliferative diabetic retinopathy undergoing SORC surgery by an experienced surgeon with no history of any ocular surgery, phakic eyes, and capable of IOLMaster examination. We excluded eyes with other retinal detachments, such as exudative retinal detachment; vitreous hemorrhage caused by other reasons, such as retinal hole, retinal vein occlusion, wet age-related macular degeneration; or any other eye diseases, keratopathy, uveitis, glaucoma, orbital tumors, eye trauma, or any history of ocular surgery. Patients who did not follow scheduled visits postoperatively were excluded.

### 2.3. Ophthalmic Examinations

Each subject was evaluated by an experienced ophthalmologist using a slit lamp (Keeler Instruments, UK). An tonometer (Topcon, Japan), B-ultrasound (Suowei, China), an ultra-wide-field scanning laser ophthalmoscope (Optos, UK), and optical coherence tomography (ZEISS, Germany) were used to further confirm the diagnosis.

### 2.4. Surgical Technique

All surgeries were performed under retrobulbar anesthesia by the same professional surgeon (Tao He) [17]. SORC surgery was performed approximately 3 months after PPV. All patients were treated with retrobulbar anesthesia. The 23G scleral trocars were made 3.5 mm from the corneal limbus, and the perfusion tube was inserted into the subtemporal trocar. The trocars were closed. A 3.2 mm incision and a two-point corneal auxiliary incision were made, and viscoelastic agent was injected into the anterior chamber. Continuous annular capsulorhexis was performed using the capsulorhexis forceps. The lens was aspirated by phacoemulsification, and a foldable intraocular lens was inserted into the capsular bag. The anterior chamber was restored by injecting balanced salt solution, and a clear corneal incision was closed. The perfusion tube was opened and the silicone oil in the vitreous cavity was removed from the scleral trocar. The scleral trocar port was then sutured with 7-0 absorbable sutures. Conjunctival sutures were removed 1 week postoperatively.

### 2.5. Refractive Analysis

The refractive status was determined using IOLMaster (ZEISS, Germany) preoperatively and at 3 days, 1 week, and 1 month postoperatively. All examinations were performed three times by a blinded observer. The following data were compiled postoperatively: best-corrected visual acuity (BCVA), refraction, intraocular pressure (IOP), retinal and macular status, and complications over a minimum 1-month follow-up period.

### 2.6. Statistical Analysis

Statistical analyses were performed using SPSS 26.0 (IBM SPSS Statistics, USA). Quantitative data are presented as mean ± standard deviation. The differences at every follow-up time point were analyzed using one-way analysis of variance with Dunnett's multiple comparison two-sided test. *P* < 0.05 was considered statistically significant.

## 3. Results

### 3.1. Demographics and Characteristic Data

We enrolled 89 eyes of 89 patients, including 41 right and 48 left eyes; 43 patients were men while 46 were women. The average age was 59.31 ± 7.57 years (range: 41–75). The primary diseases included rhegmatogenous retinal detachment and proliferative diabetic retinopathy (Table 1).

### 3.2. Comparison of Corneal Astigmatism at Different Follow-Up Time Points

The differences in K1, K2, and corneal astigmatism at the different follow-up time points are shown in Table 2. Compared to baseline, K1 decreased significantly at 3 days postoperatively (*P* = 0.016), 1 week (*P* = 0.009), and 1 month (*P* = 0.035), while K2 increased significantly at 3 days postoperatively (P = 0.002), 1 week (*P* < 0.001), and 1 month (*P* = 0.001), as well as corneal astigmatism (all *P* = 0.001).

To investigate the alterations in K1, K2, and corneal astigmatism postoperatively, we further compared each follow-up time point at 3 days postoperatively. Both K1 and K2 showed significant differences at 1 month postoperatively when compared with those at 3 days (*P*=0.029 and 0.021, respectively), while there was no statistical difference at 1 week (*P*=0.256 and 0.171, respectively). Corneal astigmatism exhibited significant differences both at 1 week and 1 month postoperatively when compared with that at 3 days (all *P* < 0.001, Table 3).

### 3.3. Comparison of BCVA, IOP, and Axial Length at Different Follow-Up Time Points

Differences in BCVA, IOP, and axial length at different follow-up time points are shown in Table 4. Compared to that at baseline, the BCVA significantly improved at 3 days, 1 week, and 1 month postoperatively (all *P* < 0.001). Meanwhile, IOP decreased significantly at 3 days postoperatively (*P* < 0.001), 1 week (*P*=0.005), and 1 month (*P*=0.007). Similarly, axial length decreased at all follow-up time points (all *P* < 0.001).

To investigate the changes in BCVA, IOP, and axial length postoperatively, we further compared each follow-up time point at 3 days postoperatively (Table 5). The BCVA showed significant differences at both 1 week and 1 month postoperatively when compared with 3 days (all *P* < 0.001), as well as the IOP (*P*=0.005 and 0.006, respectively). However, the axial length showed no statistical differences at either 1 week or 1 month postoperatively when compared with 3 days (*P*=0.653 and 0.765, respectively).

## 4. Complications

After SORC surgery, the retina remained attached in 60 eyes (96.8%), and two eyes (3.2%) in RRD patients developed re-detachment. Vitreous hemorrhage occurred in two eyes (7.4%) postoperatively in PDR patients.

## 5. Discussion

Many studies have reported on corneal astigmatism after cataract surgery. However, studies on corneal astigmatism after SORC surgery are lacking, especially in the short term. To our knowledge, this study is the first to explore short-term changes in astigmatism after SORC surgery, which has clinical significance for guiding surgical operations.

Our study found that corneal astigmatism increased significantly in the short-term postoperatively. Corneal astigmatism increased significantly 3 days postoperatively, which lasted until 1 week postoperatively, and gradually decreased 1 month postoperatively, approaching the preoperative level. Some studies on cataract surgery showed that corneal astigmatism increased significantly at one week and two weeks postoperatively, while corneal astigmatism gradually decreased at 4 weeks postoperatively, which was close to the preoperative state [18, 19]. This trend is consistent with the results of this study. The increased corneal astigmatism in the short-term postoperative period may be related to the phacoemulsification technique. With the development of phacoemulsification technology and the clinical application of folded lenses, the length of the clear corneal incision for cataract phacoemulsification technology was mostly 3.2 mm. The thermal damage of phacoemulsification and repeated warping of the inner and outer lamellae during the operation causes slight displacement of the incision [20]. Meanwhile, the radial tissue at the surgical incision is relaxed and the curvature is reduced, resulting in SIA [21, 22]. Conversely, short-term corneal edema postoperatively may cause increased corneal astigmatism. Corneal edema after cataract surgery can lead to varying degrees of curvature of the corneal surface, resulting in increased astigmatism. In this study, the degree of corneal astigmatism was the highest at 3 days postoperatively and gradually decreased at 1 week and 1 month postoperatively, which may be related to the gradual improvement of corneal edema [23].

In addition, there was some controversy regarding whether scleral tunnel incision could cause corneal SIA. Some studies suggested that simple PPV surgery will not lead to changes in corneal astigmatism [24, 25]. However, some studies also found that the steepest meridian of the cornea changed in the early postoperative 25G PPV, and the difference was statistically significant [26]. A study on vitrectomy combined with cataract surgery found that corneal astigmatism was the largest at 1 week postoperatively, and corneal astigmatism gradually recovered to preoperative levels 1 and 3 months postoperatively. In addition, the suture of the early scleral tunnel incision would cause eye discomfort and foreign body sensation, which would affect the stability of the patient's tear film and lead to poor patient cooperation during the examination. These factors may increase measured corneal astigmatism.

Some studies have suggested that the length of the eye axis changes after the removal of silicone oil. Some researchers believe that there is no statistically significant difference in axial length pre- and postoperatively [27–29]. Other researchers reported that the axial length of the eye was reduced compared with the silicone oil-filled state, which may be related to the reduction in ocular contents and intraocular pressure [30]. Elbendary and Elwan found that the refractive index shifted to hyperopia after SORC surgery [31]. In the long-term postoperative period, intraocular pressure gradually increased, resulting in an increase in axial length compared with the early stage of SOR. There was a reduction in the refractive error of hyperopia, but it still drifted towards hyperopia.

In terms of visual acuity, although the corneal astigmatism of the patients increased at 3 days and 1 week postoperatively, the postoperative visual acuity improved compared with that preoperatively. In addition, the corneal astigmatism in the two groups of patients was significantly different at 1 week and 1 month postoperatively, and the difference in visual acuity was not obvious. Visual acuity was mostly affected by the diopter reserved and condition of the fundus.

Our study had some limitations. First, we were unable to observe any longer after the SORC surgery. Meanwhile, the patient population was small, and a larger sample size should be considered in further investigations.

## 6. Conclusion

In general, SORC surgery is a safe and effective method for improving BCVA. The corneal astigmatism gradually decreases postoperatively.

## Figures and Tables

**Table 1:** Demographic and characteristic data of this study population.

	Patients	Proportion (%)
*Sex*		
Male	43	48.31
Female	46	51.69

*Eye*		
Right	41	46.07
Left	48	53.93

*Age*		
Average age	59.31 ± 7.57	—
Range	41∼75	—

*Primary disease*		
Rhegmatogenous retinal detachment	62	69.66
Proliferative diabetic retinopathy	27	30.34

**Table 2:** Comparison of corneal astigmatism status in the baseline and postoperative visit.

Parameters	Time	Mean ± SD	MD	*P* value	95% CI for difference	
					Lower bound	Upper bound
K1	Pre-op	43.20 ± 1.44	—	—	—	—
	3 D-post-op	42.76 ± 1.71	0.442	0.016^*∗*^	0.070	0.813
	1 W-post-op	42.84 ± 1.60	0.360	0.009^*∗*^	0.078	0.641
	1 M-post-op	42.95 ± 1.52	0.250	0.035^*∗*^	0.015	0.484

K2	Pre-op	44.22 ± 1.39	—	—	—	—
	3 D-post-op	44.69 ± 1.75	−0.469	0.002^*∗*^	−0.772	−0.167
	1 W-post-op	44.60 ± 1.67	−0.380	<0.001^*∗*^	−0.610	−0.150
	1 M-post-op	44.54 ± 1.62	−0.316	0.001^*∗*^	−0.512	−0.119

Corneal astigmatism	Pre-op	−1.02 ± 0.57	—	—	—	—
	3 D-post-op	−1.93 ± 1.39	0.911	<0.001^*∗*^	0.465	1.358
	1 W-post-op	−1.76 ± 1.22	0.739	<0.001^*∗*^	0.361	1.118
	1 M-post-op	−1.58 ± 1.05	0.565	<0.001^*∗*^	0.247	0.883

D, day; W, week; M, month; post-op, postoperation; SD, standard deviation; MD, mean difference; CI, confidence interval. ^*∗*^*P* < 0.05.

**Table 3:** Comparison of corneal astigmatism status in the postoperative visit.

Parameters	Time	MD	*P* value	95% CI for difference	
				Lower bound	Upper bound
K1	3 D-post-op	—	—	—	—
	1 W-post-op	−0.082	0.256	−0.211	0.047
	1 M-post-op	−0.192	0.029∗	−0.366	−0.019

K2	3 D-post-op	—	—	—	—
	1 W-post-op	0.089	0.171	−0.032	0.211
	1 M-post-op	0.154	0.021^*∗*^	0.022	0.286

Corneal astigmatism	3 D-post-op	—	—	—	—
	1 W-post-op	−0.172	<0.001^*∗*^	−0.255	−0.089
	1 M-post-op	−0.346	<0.001^*∗*^	−0.493	−0.199

D, day; W, week; M, month; post-op, postoperation; MD, mean difference; CI, confidence interval. ^*∗*^*P* < 0.05.

**Table 4:** Comparison of BCVA, IOP, and axial length in the baseline and postoperative visit.

Parameters	Time	Mean ± SD	MD	*P* value	95% CI for difference	
					Lower bound	Upper bound
BCVA	Pre-op	1.199 ± 0.565	—	—	—	—
	3 D-post-op	0.812 ± 0.403	0.387	<0.001^*∗*^	0.276	0.498
	1 W-post-op	0.751 ± 0.359	0.447	<0.001^*∗*^	0.327	0.568
	1 M-post-op	0.723 ± 0.325	0.476	<0.001^*∗*^	0.346	0.604

IOP	Pre-op	18.974 ± 3.681	—	—	—	—
	3 D-post-op	16.436 ± 3.754	2.538	<0.001^*∗*^	1.104	3.973
	1 W-post-op	17.513 ± 2.752	1.462	0.005^*∗*^	0.387	2.536
	1 M-post-op	17.667 ± 2.527	1.308	0.007^*∗*^	0.327	2.289

Axial length	Pre-op	24.543 ± 2.460	—	—	—	—
	3 D-post-op	24.347 ± 2.372	0.196	<0.001^*∗*^	0.134	0.258
	1 W-post-op	24.349 ± 2.367	0.194	<0.001^*∗*^	0.132	0.255
	1 M-post-op	24.345 ± 2.367	0.198	<0.001^*∗*^	0.136	0.259

BCVA, best-corrected visual acuity; IOP, intraocular pressure; D, day; W, week; M, month; post-op, postoperation; SD, standard deviation; MD, mean difference; CI, confidence interval. ^*∗*^*P* < 0.05.

**Table 5:** Comparison of BCVA, IOP, and axial length in the postoperative visit.

Parameters	Time	MD	*P* value	95% CI for difference	
				Lower bound	Upper bound
BCVA	3 D-post-op	—	—	—	—
	1 W-post-op	0.060	<0.001^*∗*^	0.031	0.089
	1 M-post-op	0.088	<0.001^*∗*^	0.037	0.139

IOP	3 D-post-op	—	—	—	—
	1 W-post-op	−1.077	0.005^*∗*^	−1.848	−0.306
	1 M-post-op	−1.231	0.006^*∗*^	−2.130	−0.332

Axial length	3 D-post-op	—	—	—	—
	1 W-post-op	−0.002	0.653	−0.009	0.005
	1 M-post-op	0.002	0.765	−0.004	0.007

BCVA, best-corrected visual acuity; IOP, intraocular pressure; D, day; W, week; M, month; post-op, postoperation; MD, mean difference; CI, confidence interval. ^*∗*^*P* < 0.05.

## Data Availability

The data used to support the findings of this study are available from the corresponding author upon request.
